# Supplementary material for: Are carbon emissions trading and green financial instruments synergistic? -Comprehensive quantitative research based on content analysis
Source: PLoS One. 2024 Mar 7;19(3):e0298601. doi: 10.1371/journal.pone.0298601 (PMC10919601; doi:10.1371/journal.pone.0298601)
Supplement: S1 Data — (ZIP) [file pone.0298601.s001.zip › Data/地级市Z_F工作报告.zip!/╜¡╬≈/╕╙╓▌╩╨/└┤╫╘╣1⁄2╓┌║┼í╛┬φ┐╦╩2╛▌═°í┐_2012.docx]

各位代表：

　　现在，我代表市人民政府向大会报告政府工作，请予审议，并请市政协委员和其他列席人员提出意见。

　　一、2011年工作回顾

　　一年来，在省委、省政府和市委的正确领导下，全市上下深入贯彻落实科学发展观，大力实施“新型工业化、新型城镇化、农业农村现代化、发展生态化”战略，努力建设创业、宜居、平安、生态、幸福赣州，经济社会保持平稳较快发展。突出表现在“六个历史性突破”：一是争取政策支持取得历史性突破。赣南苏区振兴发展工作得到党中央、国务院和省委、省政府的高度重视，国家层面正在抓紧研究起草支持赣南等中央苏区振兴发展的相关政策意见，11个县（市）纳入国家罗霄山集中连片特殊困难地区扶贫攻坚范围，省委、省政府明确提出构筑“龙头昂起、两翼齐飞、苏区振兴、绿色崛起”的区域发展格局，赣州发展迎来了千载难逢的重大历史机遇。二是弘扬苏区精神取得历史性突破。成功举办中央苏区革命传统主题展览、中央革命根据地创建暨中华苏维埃共和国成立80周年座谈会等纪念活动，中央首次明确提出了以“坚定信念、求真务实、一心为民、清正廉洁、艰苦奋斗、争创一流、无私奉献”为主要内涵的苏区精神。三是主要经济指标增长取得历史性突破。全市实现生产总值1335.98亿元，比年初计划多25.98亿元，增长12.5%；财政总收入180.3亿元，增长40.5%，增幅攀历史新高，创1994年分税制改革以来最好水平，位居全省各设区市首位，排位前移10位；地方财政收入110.1亿元，增长39.3%；全社会固定资产投资总额达1000亿元，增长28%，位居全省第五，排位前移5位；进出口总额29.23亿美元，增长79.31%，其中出口总额达25.23亿美元，增长92.51%，位居全省第三，排位前移7位。四是县域经济发展取得历史性突破。生产总值过100亿元、财政总收入过10亿元的县（市、区）分别达到4个和3个；超过2/3的县（市、区）财政总收入超5亿元，县县财政总收入过3亿元。五是保障性安居工程建设取得历史性突破。新建廉租住房2.1万套、公共租赁住房4.2万套，实现了人均住房建筑面积15平方米以下低收入家庭应保尽保。1.58万户农村危旧房改造和4875户“两红”人员土坯房改造全面完成。六是加强和创新社会管理取得历史性突破。持续深入开展万名干部下基层“送政策、送温暖、送服务”活动和“20万困难群众走访慰问调查”活动，为群众办好事实事10.93万件，协调处理矛盾纠纷4.23万件，解决信访问题6137件，送帮扶慰问资金2.07亿元。

　　回顾一年来的历程，我们重点推进了以下六个方面的工作：

　　（一）坚持加快发展、转型发展，转变发展方式迈出坚实步伐。

　　工业主导地位更加凸显。三次产业结构由18.9︰44.4︰36.7调整为17.4︰47.2︰35.4。三个千亿元产业集群建设扎实推进。全市规模以上工业增加值430亿元，比年初计划多80亿元，增长18.7%；主营业务收入、利税总额分别达1841.6亿元和212.9亿元，增长53.8%和91.4%。稀土和钨及其应用产业主营业务收入达640亿元，增长93.5%。57个投资亿元以上项目竣工投产或部分投产。主营业务收入过亿元或利税总额超千万元企业达到465户，比上年增加78户。全市工业园区主营业务收入1654.9亿元，过100亿元的园区达到7个。

　　农业基础地位更加巩固。实现农业总产值375亿元，增长4%。粮食总产量278.5万吨。六大农业主导产业快速发展。成功承办第三届中国赣州国际脐橙节，“赣南脐橙”成为中国驰名商标。脐橙种植面积达174万亩，产量突破133万吨。新增国家级农业标准化示范区3个、无公害农产品26个。新增年销售收入500万元以上农业龙头企业30家。完成农民技术培训20万人次。实施213座病险水库除险加固，新增、恢复和改善灌溉面积51.57万亩。启动高标准农田建设16万亩，实施土地整理8.89万亩。防汛抗旱减灾成效显著。

　　现代服务业更加繁荣。社会消费品零售总额432亿元，增长17.3%。区域性金融中心、物流中心、旅游中心建设加速推进。兴业银行、海通证券等金融机构进驻赣州，小额贷款公司实现县域全覆盖，赣州稀有金属交易所挂牌成立，组建赣州农村商业银行获国家银监会批复。居民储蓄存款和贷款余额分别达到1100亿元和1035亿元。特大型综合物流园区项目正式签约，投资总额超过100亿元。天虹、大润发等大型连锁购物中心相继入驻。赣州黄金机场进出港人数达到51.5万人次。新增4A级景区5个。瑞金、兴国两个国家级红色旅游标准化试点项目通过验收。

　　（二）基础设施加速完善，城乡面貌显著改观。开工建设重点工程155个，完成年度投资493.5亿元，比上年增加123.3亿元。开工续建高速公路304.8公里，隘瑞、瑞寻高速公路建成通车。完成国省道改造217公里。中心城区开工城建项目86个，完成投资79.4亿元，建成区面积扩大到85.23平方公里。赣南大道等一批城市重点项目建成。新购置公交车105辆，环卫作业车99辆。 “治脏、治乱、治堵”工作取得明显成效。文清路商业街综合改造、“百街小巷”整治和中心城区亮化绿化样板路三大工程，得到了市民的理解、拥护和赞同，城区环境更加优美，街道更加整洁，通行更加顺畅。统筹城乡发展试验区建设加快推进。完成1739个村庄整治工作，启动6247个村点垃圾无害化处理工作。新建农村公路1799公里，符合通行条件的行政村通客车率93.4%。

　　（三）改革开放深入推进，发展活力竞相迸发。全面完成非工口七个系统国有企业改革。国库集中支付范围不断扩大，市直预算单位全面推行公务卡管理。启动了事业单位改革。医药卫生体制改革扎实推进。小微型企业服务机制不断完善，民营经济加快发展。全市个私企业户数达23.27万户，非公有制经济实现增加值占比达60.4%，实缴税金占全市税收比重达70%。开放型经济保持良好发展态势。实际利用外资9.29亿美元，增长11.1%；引进省外5000万元以上项目157个，实际进资306.96亿元，增长30.52%。

　　（四）切实增进民生民享，民生保障水平明显提高。全年民生类财政支出169.6亿元，占总支出的54.4%。100件民生实事基本完成。新增城镇就业11.67万人，城镇就业率达96.8%。再就业小额担保贷款发放总量继续保持全国地级市首位。在全省率先实现城乡居民社会养老保险制度全覆盖。城乡低保、农村五保供养和医疗救助标准进一步提高。新农合参合率达96.37%。公办基层医疗卫生机构全面规范实施国家基本药物制度。“光明·微笑”工程扎实推进。建立居民健康档案140万份。高龄老人生活补贴发放范围扩大到80周岁以上。新解决47.62万农村居民和5.54万农村学校师生饮水安全问题。完成1119个省扶贫困村整村推进年度扶贫开发任务，移民搬迁21886人。居民消费价格指数上涨4.9%，分别低于全国、全省0.5和0.3个百分点。城镇居民可支配收入16058元，农民人均纯收入4684元，分别增长13.1%和12%。

　　（五）生态建设成效显著，各项事业协调发展。大力推进发展生态化战略。完成造林绿化89.92万亩，全市森林覆盖率提高至76.25%。赣江源晋升为国家级自然保护区。新增湿地公园面积16万亩。实施小流域水土保持综合治理82条，治理水土流失面积96万亩。完成废弃稀土矿山水土保持综合治理1915亩，废弃矿山复绿2万亩。扎实开展农业面源污染治理。主要河流国、省、市断面水质达标率91%，环境空气质量优良率100%，主要污染物总量减排完成省下达任务。

　　科技创新“六个一”工程取得新成效，新增国家级创新型企业1家、高新技术企业7家。专利申请量和授权量分别增长73.9%和67.2%。新增省级教育园区建设试点县（市）8个。成功申报创建第一批国家公共文化服务体系示范区。新建农家书屋821个。大型采茶歌舞剧《八子参军》获国家舞台艺术精品工程年度资助剧目奖。圆满完成“七城会”相关赛事承办工作，成功举办市三运会。在全国设区市中率先设立红十字博爱基金。人口计生工作水平不断提高，人口自然增长率7.52‰。精神文明建设进一步深入，成功跻身第三批全国文明城市提名资格城市。再创全国“双拥”模范城。民主法治建设得到加强。新闻出版、外事侨务、民族宗教、涉台事务、气象测绘、防震减灾、统计、审计、供销、监察、司法行政、检验检疫、工商、质量监督、食品药品监管、民兵预备役、国防动员、人民防空、方志档案、妇女儿童、老龄、慈善、残疾人等各项工作取得新成绩。安全生产形势进一步好转，社会治安综合治理得到加强。

　　（六）强力推进“治庸、治懒、治散”，政府自身建设进一步加强。在市委的统一领导下，顺利完成市、县、乡三级政府换届。继续推进行政审批制度改革，取消或下放非行政许可审批事项41项，网上审批和电子监察系统进一步完善。发展提升年活动获全省先进。坚持依法行政，加强重大决策和政策文件合法性审查，强化行政复议和执法监督。自觉接受人大、政协监督，办理人大代表建议、政协提案共535件，交办率、办结率、满意和基本满意率均达100%。应急管理机制不断完善，突发事件应对和处置能力有新的提升。开展医药、教育、涉农收费、保障房建设等领域专项治理工作，反腐倡廉、惩治和预防腐败体系建设取得新成效。

　　各位代表，一年来，在以文清同志为班长的市委高端谋划、高度重视、高位推动下，我们历史性地启动了推动赣南苏区振兴发展工作，深入开展全面系统的课题调研，建立详细的数据台账，策划、提出了200多个重大项目，明确了赣南苏区振兴发展的思路和方向。大范围、深层次、高密度地向中央领导和国家有关部委汇报对接，积极争取中央和社会各界聚焦、支持赣南苏区发展。目前，国家部委联合调研组来赣州展开大规模调研的各项准备工作已经就绪，推动赣南苏区振兴发展上升为国家战略到了加油冲刺的阶段。

　　各位代表，回顾一年来的工作，成绩令人鼓舞。这是市委准确把握发展大势、高瞻远瞩科学决策、统揽全局正确领导的结果，是市人大和市政协监督支持、共同努力的结果，是全市人民齐心协力、团结奋斗的结果。在此，我代表市人民政府，向全市广大工人、农民、知识分子、干部和历任老领导、老同志，向各民主党派、工商联、无党派和社会各界人士，向驻市部队、武警官兵和公安干警，致以崇高的敬意！向港澳同胞、台湾同胞和海内外友好人士，以及所有关心支持赣州发展的同志们、朋友们，表示衷心的感谢！

　　在看到成绩的同时，我们也清醒地认识到存在的困难和问题。主要表现在：经济发展依然滞后，后发展、欠发达的基本市情没有根本改变；产业基础差、规模小、层次低，重大项目支撑不够；基础设施建设滞后，发展后劲不足的矛盾依然比较突出；资源、环境、要素趋紧，转变经济发展方式任务繁重；人民生活仍然比较困难，改善民生方面还有不少问题亟待解决；政府部门干部作风建设有待进一步加强。对此，我们将采取更加有力的措施，努力加以解决。

　　二、2012年工作的总体要求和目标

　　2012年是赣州加快发展、转型发展的关键之年。做好今年工作任务繁重，意义重大。尽管宏观经济形势依然复杂严峻，但我国经济保持平稳较快增长的基本面没有改变。国家继续实施积极财政政策和稳健货币政策，特别是提出深入推进新一轮扶贫开发，出台帮扶政策大力支持赣南苏区振兴发展，省委、省政府将着力构筑“龙头昂起、两翼齐飞、苏区振兴、绿色崛起”的区域发展新格局，为我们加快发展、转型发展提供了千载难逢的机遇。我们一定要认清形势，把握大局，坚定信心，在“稳中求进”的基础上，好中求快，又好又快，努力实现赣州经济快速、健康、协调和可持续发展。

　　今年政府工作的总体要求是：以邓小平理论和“三个代表”重要思想为指导，深入贯彻落实科学发展观，全面贯彻中央、省经济工作会议和省第十三次党代会、市第四次党代会精神，紧紧围绕打造全省发展重要增长极、特色产业聚集区、绿色发展先行区、和谐发展示范区和作风建设模范区，以赣南苏区振兴发展为契机，突出抓项目、扩投入，兴产业、调结构，惠民生、促和谐，努力实现经济快速增长、民生持续改善、社会和谐稳定，推动加快发展、转型发展取得实质性进展，加快建设创业、宜居、平安、生态、幸福赣州，以优异成绩迎接党的十八大胜利召开。

　　2012年，我市经济社会发展的主要预期目标是：生产总值增长12%；财政总收入增长20%；全社会固定资产投资增长22%；规模以上工业增加值增长18%；社会消费品零售总额增长18%；实际利用外资增长10%；进出口总额增长5%，其中出口增长5%；城乡居民收入分别增长13%以上；居民消费价格涨幅控制在4%左右；人口自然增长率控制在7.58‰以内；单位生产总值能耗下降3.26%，化学需氧量和氨氮排放量分别下降1%。

　　做好今年工作，完成上述目标任务，必须坚定不移地以赣南苏区振兴发展激活经济社会发展全局，调动各方力量，凝聚各方智慧，积极作为，扎实努力，推动赣南苏区振兴发展上升为国家战略，确立“南有中央苏区、北有鄱阳湖区”的战略地位；必须坚定不移地以项目建设为抓手，重点围绕建设区域性综合交通枢纽、壮大“三个三”产业集群、建设三大区域性中心谋划实施一批重大项目，扩大投资规模、优化投资结构、拓宽投融资渠道，为赣南苏区振兴发展奠定坚实基础；必须坚定不移地以产业建设为核心，打造一批特色产业园区和特色产业基地，培育一批航母型产业、旗舰型企业，壮大有赣州特色的产业集群，以产业的强势发展带动全市经济的强势崛起；必须坚定不移地以新型城镇化为动力，进一步完善城市功能，拉开城市框架，提升城市品质，以特大型中心城市和赣南城市群的迅速壮大，拉动全市经济的快速发展；必须坚定不移地以保障和改善民生为出发点和落脚点，加快建设创业、宜居、平安、生态、幸福赣州，让更多的阳光政策普惠群众，更多的发展成果惠及群众；必须坚定不移地按照市委既定的发展思路、发展理念和发展战略，心无旁骛抓执行抓落实，凝心聚力谋发展，果敢担当破难题，以各项工作的提速提质提效开创赣州加快发展、转型发展的新局面。

　　三、以赣南苏区振兴发展为契机，推动加快发展、转型发展取得实质性进展

　　（一）全力以赴推动赣南苏区振兴发展

　　把加快赣南苏区振兴发展作为谋全局管当前利长远的头等大事。全力争取国家尽快出台支持赣南苏区振兴发展的政策意见。进一步深化市情认识，找准制约发展的症结和瓶颈，深入研究赣南苏区振兴发展的思路举措。加强与国家有关部委和省有关部门的沟通对接，主动自觉地做好配合国家有关部委实地调研等基础性工作。凝聚赣南苏区振兴发展的强大合力。大力弘扬苏区精神和优良革命传统，推动苏区精神、苏区历史、苏区干部好作风进机关、进农村、进学校、进企业、进社区；积极组织开展宣传苏区精神和红色文化的各类活动，切实把智慧和力量凝聚到赣南苏区振兴发展上来。对接跟进赣南苏区振兴发展的重大项目。紧扣赣南苏区振兴发展的政策意见和相关对接事项，策划和包装一批群众热切期盼的重大民生项目、增强“造血”功能的重大产业项目、积蓄发展后劲的重大基础设施项目。尤其要注重策划包装一批影响赣州经济社会长远发展的重大项目。做好昌吉赣客专并南延至深圳、鹰瑞梅铁路、华能瑞金电厂二期工程等项目前期工作，争取尽早开工。加快重点工程建设进度。今年，全市安排重点工程建设项目168个，年计划投资608.86亿元。力争赣韶铁路，大广高速龙杨段，厦蓉高速赣崇段，赣州220千伏、110千伏输变电工程，省天然气二期管网，中心城区天然气管网等项目年内建成；黄金机场改扩建、寻全高速公路、兴国至赣县高速公路、593公里国省道改造、江西华电赣州开发区天然气利用、红都瑞金500千伏输变电工程等项目年内开工建设。真正让中央的关怀转化为一个个具体项目，让国家的扶持转化为坚持加快发展、推动转型发展的强劲动力，让赣南苏区振兴发展的伟业转化为全体赣南人民的共识共为。

　　（二）着力推进新型工业化

　　始终坚持强攻工业、主攻工业，采取一切可能的措施，保持工业发展的强劲势头，挺起赣南苏区振兴发展的脊梁。

　　做大做强产业集群。以“三个三”产业为主抓手，推动矿产资源产业高端化、战略性新兴产业规模化、承接产业品牌化、传统产业高新化。坚持“政府引导、市场运作”，全面推进稀土、钨、萤石资源整合。落实国家稀土产业发展政策，加强与央企和科研院所合作，加快组建以赣州稀土集团公司为主体的南方大型稀土集团。加大矿产勘查开发利用与保护力度，推进稀土开采转型，加快推进稀土等矿山法人开采，建设标准化稀土矿山。争取国家设立稀土、钨专项储备金，将赣州列入储备试点和找矿突破战略行动重点区域。充分发挥赣州稀有金属交易所的作用，打造稀土、钨交易中心和集散中心。抓紧建设提升国家级钨和稀土工程研究院、工程技术研究中心、检验检测中心等科研平台。出台分类扶持措施，大力推进铜铝有色金属产业、新能源汽车及其配套产业以及氟盐化工、电子信息、现代轻纺、食品工业、新型建材、生物制药、家具等产业上规模、上水平。加大力度帮扶企业。设立战略性新兴产业发展基金，支持高科技企业发展。鼓励企业与高校、科研机构合作，推动产学研合作科技成果产业化。鼓励央企与地方企业对接合作、对口帮扶、联合重组。

　　加强工业园区建设。进一步完善产业规划布局，加快赣州开发区扩区增容，申报国家级循环经济试点园区、国家生态工业示范园区。争取在瑞金、兴国设立特殊经济区。支持出口加工区转型升级为国家综合保税区。推动瑞金、龙南和赣县经济技术开发区升级为国家级开发区，龙南、定南、全南建设国家级承接产业转移示范区，寻乌、石城和崇义设立省级工业园区。积极与深圳等沿海地区合作共建产业园区。加强园区交通、通信、供水、供气、供电、防灾减灾、污水治理等设施建设，增强园区综合配套能力。实施产业基地公共服务能力提升工程，提高管理效率和服务水平，增强承载能力，打造主业突出、特色鲜明的园区板块。高位启动稀土高新产业园、铜铝产业园建设。发展壮大南康家具、龙南发光材料、信丰电子信息、赣县钨稀土和铜铝新材料、会昌氟盐化工等特色产业园区。

　　（三）着力推进中心城市建设

　　把城市扩容增量，加快提高中心城市首位度，提升城市集聚力和辐射力作为战略重点，以中心城市崛起打造赣南苏区振兴发展的核心增长极。

　　做大城市规模。编制实施《赣州特大城市城乡总体规划》，强化产业集聚效应，提高空间拓展效能，提升生态文化品质，分类引导项目建设，优化行政区划管理。推动赣县、南康、上犹与主城区在基础设施、综合交通、生态环保、产业布局等方面全方位对接、同城化发展。加快章江新区繁荣发展，推进老城区改造，协调各组团加快建设。争取南康撤市设区，力争年内中心城区建成区面积过100平方公里、人口过100万，迈入特大城市方阵。抓好100个重点城建项目建设。进一步完善章江新区等各组团内部路网，打通文明大道东延等8条断头路、瓶颈路。开工建设“四桥”及“四纵五横”路网①，打通二环线②、内环线③，构建“两快一慢一枢纽”④的交通网络体系，全面拉开特大城市框架。

　　提升城市功能品质。加快建设中心城区截污干管、排水管网、第二污水处理厂、第二垃圾处理场，启动上犹江引水工程前期工作，提速配套市政公用设施。加快自然博物馆、文化艺术中心、奥体中心、会展中心、华润万象城城市综合体等项目建设，提升城市公共服务设施水平。深入推进“百街小巷”综合整治，建成东阳山路和八一四大道人行天桥。加快建设公共停车场。新购100辆公交车，建设100个公交候车亭，新增赣南大道等5条公交线路，开辟公交专用车道和快速公交线，优化线路设置，延长运营时间。进一步增加出租车数量，规范出租车运营秩序。继续完成文清路改造，开工并完成郁孤台、八境台历史文化街区改造，打造具有宋城印象的城市名片。持续深入推进中心城区“治脏、治乱、治堵”工作，完善城管机制，创新城管手段，提升环境净化、园林绿化和美化亮化水平。

　　（四）着力推进农业农村现代化

　　坚持农业基础地位不动摇，落实好各项强农富农惠农政策，不断提高农业生产能力，促进农民增收，改善农村生产生活环境。

　　大力发展现代农业。进一步巩固粮食基础地位，力争粮食总产量280万吨以上。进一步加强赣南脐橙品牌建设和质量标准体系建设，巩固和完善市场营销体系，办好第四届国际脐橙节。建设农产品信息服务中心、国家级脐橙交易中心、赣南脐橙研究院，组建赣南脐橙股份制公司并推进上市。进一步培育壮大生猪、烟叶、花卉苗木、蔬菜、油茶、茶叶等主导产业，打造沿海重要的优质农产品供应基地。高起点、高标准创建一批现代农业产业示范园区，启动国家级现代农业高新产业园建设。加强农业科技创新推广，加快建立现代农业产业技术体系。大力引进和培育一批产业关联度高、品牌知名度高、科技含量高的农产品精深加工企业。积极发展农业专业合作组织，壮大农产品营销队伍和农村经纪人队伍。建设农村现代流通服务体系，抓好农超对接，建立和完善农产品市场信息联接机制，搞活农产品流通。加大农民培训力度，提高农民素质。

　　加强农业农村基础设施建设。下大力气实施水库保安和农村饮水安全“双安”工程，明年汛前完成669座小（2）型病险水库除险加固；制定实施“农村饮水安全工程三年规划”，力争两年完成。实施23个中小河流治理项目。抓好16万亩高标准农田建设。大力实施农村土地整治、城乡建设用地增减挂钩试点，积极推进低丘缓坡荒滩等未利用土地开发利用试点、工矿废弃地复垦和调整利用试点。新开工建设农村公路1500公里，改造农村公路危桥120座，力争符合条件的行政村通客车率达95%，实现县乡村公路养护全覆盖。扎实推进新农村建设，抓好1703个省批村点的村庄整治建设，推进3747个村点农村清洁工程，新建农村无害化卫生户厕4.7万座。实施好新一轮农村电网升级改造和户户通电视工程，确保农民能喝上洁净的水、用上电、看上电视。

　　抓好新一轮扶贫开发。建立以公共财政为主的扶贫开发投入体制。市财政整合1亿元资金，专项用于扶贫开发。编制并启动罗霄山特困片区11个县（市）区域发展与扶贫攻坚规划。采取每位市党政领导带一个市直（驻市）部门、一个市属企业重点帮扶一个县、抓一个示范乡镇和一个示范村，市直县直单位定点扶持贫困村的做法，大力推进片区扶贫、移民扶贫、整村扶贫、产业扶贫和素质扶贫。积极探索开发式扶贫与救助性扶贫相结合的新模式，多渠道筹措扶贫资金。市级设立扶贫救助基金，投资标准厂房建设、土地收储和入股优势企业，基金收益用于农村五保对象和10万特困人口的扶贫救助，让特殊困难群体“不愁吃、不愁穿”。

　　（五）着力推进现代服务业发展

　　促进服务业与制造业有机融合，与新型城镇化互动发展，吸引人流、物流、资金流、信息流向中心城市集聚，为赣南苏区振兴发展培育新的经济增长点。

　　加快区域性金融中心建设。启动金融商务核心区建设。大力引进域外金融机构，做大做强赣州银行等地方金融机构，争取组建市级统一法人的农村商业银行，推动村镇银行、小微企业贷款服务中心县域全覆盖，有序发展小额贷款公司和融资性担保公司。引导金融机构创新金融产品和服务方式，加大对实体经济，对重点项目、重点产业、小微型企业和“三农”的信贷支持力度。积极推动企业上市，在资本市场打造“赣南板块”。大力推进社会信用体系建设，优化金融生态环境，切实防范金融风险。

　　加快区域性物流中心建设。争取列入全国流通领域现代物流示范城市，开展农产品现代流通综合试点。加快推进赣州综合物流园区建设，打造成区域性工业原料及成品交易中心和现代综合商贸物流城。加快航空港综合物流中心等六大物流节点建设。培育和引进核心竞争力强、主营业务突出的现代物流企业，着力壮大电子物流、大件物流、有色冶金物流、冷链物流。推进物流信息平台与标准化建设，提升物流企业现代化管理水平和市场竞争力。

　　加快区域性旅游中心建设。加快瑞金红色旅游景区、通天岩景区创5A步伐，抓好陡水湖风景区开发、宋城文化遗址公园、龙南客家围屋博物馆等项目建设，策划包装好中央苏区红色旅游和三江六岸、百里脐橙旅游观光带等重点项目。培育旅游龙头企业和著名品牌。完善旅游公路、景区景点等基础设施，提升配套功能。举办中国赣州生态文化旅游节，形成富有红色、客家、宋城、生态等特色的旅游产品体系，建设全国著名的旅游目的地。

　　（六）着力推进改革开放

　　坚持以改革增活力、以开放借外力，下大决心和气力解决制约发展的体制性、结构性矛盾，不断提升对外开放的层次和水平，为赣南苏区振兴发展汇聚持续强大的力量源泉。

　　深化各项改革。进一步清理、取消和调整行政审批事项。加强行政服务中心、便民服务中心、公共资源交易中心、投诉中心建设，建立政府部门服务企业发展、服务项目建设、服务县域经济和园区发展的长效机制。抓好事业单位改革。推进收入分配制度改革。深化文化体制改革，推动经营性文化单位转企改制。加大国企改革力度，建立健全法人治理结构，引进战略投资者，鼓励国有企业靠强联大，增强市场竞争力。出台扶持民营企业加快发展、返乡创业人员投资创业的优惠政策。整合政府资源和国有资产、资本，组建国有控股投融资集团、金融控股公司和赣州金融资产交易中心，加强政府性融资平台管理。积极稳妥推进国有林场和集体林权制度配套改革，组建林业投资集团，建设林权交易中心。推进农村金融服务改革，加大农业保险和农房保险力度，加快农村集体土地确权登记发证工作。

　　大力招商引资。改进和完善招商引资考评办法，强化激励机制，重奖对招商安商有重大贡献的单位和个人。创新招商引资方式，瞄准国际国内500强、中央和省属大企业、优质上市公司、大型民营企业，开展高端谋划，高位推动，高频对接。大力推进产业招商，重点在龙头企业招商、配套产业招商、成建制承接产业转移上取得新突破，争取列为“全国加工贸易产业转移示范地”。办好“共和国央企回故乡”座谈会、香港招商引资活动周、赣州（厦门）投资环境推介会等重大活动，打造招商引资新平台。

　　发展民营经济。落实关于鼓励引导民间投资新36条和支持小微型企业、个体工商户发展的各项优惠政策，加大指导和支持服务力度，减轻企业税费负担，强化金融支持，改善民营企业特别是小微型企业生存发展环境。力争年内新增个体工商户1万户、私营企业3000家，培育300家高成长性企业上规模，帮扶100家“专、精、特、新”企业创品牌，全市民营经济增加值突破1000亿元。

　　（七）着力推进县域经济发展

　　坚持以县（市、区）为基本单元，进一步“放权、让利、松绑”，打造县域经济“动车组”，为赣南苏区振兴发展强筋健骨，使县域经济成为重要的经济增长点。

　　壮大县域经济实力。充分挖掘和放大各地的比较优势，引导各县（市、区）走差异化特色化发展之路，培育各具特色的县域经济板块。出台加快县域经济发展的实施意见，在财政、金融、人才、科技、用地等方面加大支持力度，继续加强县域基础设施建设，不断改善县域经济发展条件。对县域经济发展实行分类考核，加大奖励力度，鼓励争先进位，力争GDP过100亿元、财政总收入过10亿元的县（市）分别新增3个和5个。

　　增强县域经济发展活力。推进扩权强县、兴乡强镇，最大限度下放管理审批权限。建立健全服务县域经济发展新机制，综合运用市领导挂点帮扶、市直部门点对点服务、重大问题现场办公等方式，帮助解决瓶颈问题。充分调动乡镇发展经济的积极性，鼓励乡镇走出去招商引资，打造一批实力乡镇。

　　提升县域城镇化水平。加快瑞金、龙南都市区建设，加快信丰、兴国、于都、宁都等卫星城市建设，打造组合有序、层次分明、协调发展的赣南城市群。加快以示范镇为重点的圩镇建设，培育一批产业重镇、商贸强镇、旅游名镇。抓好储潭、三江及各县（市）统筹城乡发展示范区建设。积极探索统筹城乡发展支撑体系，逐步建立城乡统一的户籍制度，推动公共资源城乡均衡配置、生产要素城乡自由流动，促进城乡基本公共服务均等化。

　　四、以改善民生为重点加快发展各项事业，努力建设创业、宜居、平安、生态、幸福赣州

　　坚持把保障和改善民生作为最大的政治、最大的责任，让更多的发展成果实实在在装进老百姓的口袋。

　　抓好民生工程建设。今年，全市筹资200亿元以上，集中办好100件民生实事。为切实增强民生实事的实效性、群众性，市委、市政府在多次反复广泛征求意见的基础上，又首次通过媒体再次征求全市广大市民的意见。重点是：落实扶持就业创业政策，力争城镇新增就业6.06万人，新增转移农村劳动力9.59万人，新增发放小额担保贷款6亿元；推进城乡居民社会养老保险全覆盖，实现城镇职工基本医疗保险市级统筹；进一步提高城乡低保、农村五保供养、大病医疗救助等社会救助标准，提高城镇居民基本医疗保险和新农合补助标准，提高城镇职工和居民医疗保险待遇；新（改、扩）建“三院”（敬老院、光荣院、福利院）36个，扶持每个县（市、区）新建一个示范性社区服务中心和一个示范性居家养老服务中心；继续为白血病和先天性心脏病患儿实施免费救治，为困难尿毒症患者免费透析，为农村65岁以上老人免费进行健康体检，为肢体残疾人免费安装假肢，为妇女“两癌”（乳腺癌和宫颈癌）患者在市定点医院免费实施手术；抓好“菜篮子”工程建设，中心城区新增4000亩蔬菜基地，实施好放心早餐、放心快餐和放心豆制品生产工程；加强民生价格监管，严厉查处乱收费行为，确保物价涨幅不超过全国全省平均水平。

　　扎实推进保障性安居工程建设。继续落实房地产市场宏观调控政策，增加商品房有效供给，促进房价合理回归。创新保障性住房建设模式，完善准入、分配、管理、退出制度，推进廉租住房、公共租赁房、经济适用房“三房合一”、并轨建设、租售并举，落实质量安全监管“三个百分百”⑤。把人才公寓纳入公共租赁房建设范围，年内建设各类保障性住房4.7万套，其中廉租住房13570套、公共租赁住房21887套、各类棚户区改造1.16万户。启动实施农村土坯房改造试点工作，优先解决革命烈士、“两红”人员、在乡老复员军人后代及家属土坯房问题。

　　加快各项事业发展。深入推进科技创新“六个一”工程，围绕重点产业发展，实施一批重大科技项目，布局一批科技创新平台，打造一批科技创新团队，扶持一批拥有自主知识产权、发展前景好的企业。推进区域性教育和医疗服务中心建设。实施农村义务教育学生营养改善计划试点。继续抓好中小学、幼儿园布局规划调整，加快教育园区、职教园区和示范性综合实践基地建设步伐，完成25万平方米中小学校D级危房拆除重建任务，新（改、扩）建100所乡镇公办幼儿园。加强校车安全管理，创建和谐平安校园。进一步加强城乡医疗卫生服务体系建设，在行政村卫生室推行国家基本药物制度，提高医疗机构服务水平。以国家公共文化服务体系示范区创建工作为抓手，推进文化基础设施升级提质。免费开放博物馆、纪念馆、图书馆、文化馆。启动江南宋城核心区规划，抓好赣南客家围屋申报世界文化遗产工作，做好赣南客家文化生态试验区申报工作，推进革命旧居旧址抢救性保护工程。加快发展红色文化产业，打造一批苏区文化品牌。继续实施计划生育奖励扶助和特别扶助制度，切实稳定低生育水平，综合治理出生人口性别比偏高问题，加快实现计划生育优质服务全覆盖。深入开展全民健身活动，加快全民健身的体育场馆建设。广泛开展全民国防教育，大力加强国防后备力量建设。

　　加强生态环境保护。完成造林绿化70万亩，实施低产低效林改造工程，抓好公益林保护。推动建立和完善生态补偿机制。实施钨和稀土资源利用生态化示范工程、废弃矿山综合治理等生态项目，完成2万亩废弃稀土矿山复绿任务。加强生态环境综合治理与保护，完成40条小流域水土保持综合治理，加强农业面源污染治理和农村垃圾无害化处理，确保主要河流监测断面水质达标率在90%以上，环境空气质量优良率保持100%。积极争取列入国家节能减排财政政策综合示范城市，严格落实环境保护和节能减排目标责任制，确保完成省政府下达的主要污染物减排任务。

　　着力维护和谐稳定。以推动干部下基层“送政策、送温暖、送服务”工作全覆盖、常态化为总抓手，加强和创新社会管理，做好新形势下群众工作。抓好一批标准化社区建设。做好流动人口和重点特殊人群服务管理。加强“两新”组织⑥和网络虚拟社会管理。认真排查治理安全生产隐患，坚决遏制重特大安全事故发生。推进食品药品安全示范区建设，健全监管网络，提升食品药品安全保障水平。健全突发事件预防预警和应急处置机制。加大地质灾害防治力度，做好防大汛、抗大旱的准备。抓好“天网”工程建设，实现城区监控全覆盖。强化社会治安综合治理，依法严厉打击各类犯罪活动。建立健全人民调解、行政调解、司法调解“三调”联动工作机制，妥善化解矛盾纠纷。开展信访积案化解攻坚年活动，做好信访维稳工作，不断提高人民群众的安全感和满意度。

　　五、以提高推动加快发展、转型发展的能力为重点，切实加强政府自身建设

　　做好2012年的工作，推动赣南苏区振兴发展，对政府工作提出了新的更高的要求，我们必将知责负重，自我加压，踏实干事，积极作为，努力开创政府工作新局面。

　　提高落实执行的能力。继续大力“治庸、治懒、治散”，推动政府工作提速提质提效。努力把市第四次党代会的决策部署进一步落实到具体举措，把加快发展、转型发展的任务落实到具体项目，把建设创业、宜居、平安、生态、幸福赣州的要求落实到具体部门。全力打好发展建设中的硬仗，积极化解阻碍改革发展的坚冰，努力突破事关加快发展、转型发展的瓶颈。

　　提高科学施政的能力。继续完善政府班子学习制度，对宏观形势多分析多思考，对热点问题多关注多研究，对重大事项多调研多论证。不断创新政府工作理念、方法和机制，工作谋划重长远讲科学，工作推动重细节讲程序，工作落实重结果讲效率。建立健全维护和保障群众利益决策机制，健全重大决策专家论证、风险评估和社会公示制度，主动按照议事规则和程序作决策，主动遵循市场经济规律促发展，主动尊重群众意愿办实事。

　　提高依法行政的能力。推进法治政府建设，进一步规范重大决策和政策文件制定程序，强化行政复议和执法监督，化解行政争议和社会矛盾。认真落实市人大及其常委会各项决议决定，自觉接受人大的法律监督和政协的民主监督，切实提高建议、提案的办理质量。充分听取各民主党派、工商联、无党派人士以及各人民团体的意见和建议，广泛接受新闻舆论和社会公众监督。加强重大政策文件备案审查，做到有件必备、有错必纠。加大政府信息公开力度，及时回应社会关切，正确引导社会舆论。

　　提高拒腐防变的能力。开展创建作风建设模范区暨集中整治影响发展环境的干部作风突出问题活动，民主评议政风行风，促进作风大改进、大提升。树立用制度、科技、综合手段治腐的理念，认真落实党风廉政建设责任制，深入推进惩治和预防腐败体系建设。加强对重点领域、重点部位和关键环节的权力运行监管，全面规范公共资源招标投标市场，完善政府采购、产权交易、项目招投标等制度。深化电子政务和电子监察系统建设，逐步实现政务在网上公开、公共资源在网上交易、监督在网上实施。对市政府门户网站升级改版，开通网络问政平台。认真执行《廉政准则》，严格控制公务购车、出国出境、会议接待、庆典活动等支出，厉行节约、勤俭办事，努力建设一支作风过硬、廉洁奉公、人民群众满意的公务员队伍。

　　各位代表，征程万里风正劲，重任千钧须奋蹄。完成2012年各项目标任务，意义深远，责任重大，使命光荣。让我们更加紧密地团结在以胡锦涛同志为总书记的党中央周围，深入贯彻落实科学发展观，在市委的正确领导下，始终保持昂扬向上的锐气，敢立潮头的勇气，提振精神，求真务实，奋发有为，全力推动赣南苏区振兴发展，以优异成绩迎接党的十八大胜利召开！
